# BI 1291583: a novel selective inhibitor of cathepsin C with superior in vivo profile for the treatment of bronchiectasis

**DOI:** 10.1007/s00011-023-01774-4

**Published:** 2023-08-04

**Authors:** Stefan Kreideweiss, Gerhard Schänzle, Gisela Schnapp, Viktor Vintonyak, Marc A. Grundl

**Affiliations:** Boehringer Ingelheim Pharma GmbH & Co. KG, Biberach, Germany

**Keywords:** Preclinical, Cathepsin C inhibitor, Bronchiectasis, Neutrophil-derived serine protease activity

## Abstract

**Background:**

Airway inflammation in chronic inflammatory lung diseases (e.g. bronchiectasis) is partly mediated by neutrophil-derived serine protease (NSP)/antiprotease imbalance. NSPs are activated during neutrophil myelopoiesis in bone marrow by cathepsin C (CatC; DPP1). CatC is therefore an attractive target to reduce NSP activity in the lungs of patients with bronchiectasis, restoring the protease/antiprotease balance. We report results from the preclinical pharmacological assessment of the novel CatC inhibitor BI 1291583.

**Methods:**

Binding kinetics of BI 1291583 to human CatC were determined by surface plasmon resonance. In vitro inhibition of human CatC activity was determined by CatC-specific fluorescent assay, and selectivity was assessed against related cathepsins and unrelated proteases. Inhibition of NSP neutrophil elastase (NE) production was assessed in a human neutrophil progenitor cell line. In vivo inhibition of NE and NSP proteinase 3 (PR3) in bronchoalveolar lavage fluid (BALF) neutrophils after lipopolysaccharide (LPS) challenge and distribution of BI 1291583 was determined in a mouse model.

**Results:**

BI 1291583 bound human CatC in a covalent, reversible manner, selectively and fully inhibiting CatC enzymatic activity. This inhibition translated to concentration-dependent inhibition of NE activation in U937 cells and dose-dependent, almost-complete inhibition of NE and PR3 activity in BALF neutrophils in an in vivo LPS-challenge model in mice. BI 1291583 exhibited up to 100 times the exposure in the target tissue bone marrow compared with plasma.

**Conclusion:**

BI 1291583-mediated inhibition of CatC is expected to restore the protease–antiprotease balance in the lungs of patients with chronic airway inflammatory diseases such as bronchiectasis.

**Supplementary Information:**

The online version contains supplementary material available at 10.1007/s00011-023-01774-4.

## Background

Bronchiectasis is a disease of the lower respiratory tract that is caused by a wide range of underlying clinical disorders [1]. Underlying aetiologies range from well-characterised genetic diseases such as cystic fibrosis (CF) and primary ciliary dyskinesia, to asthma, chronic obstructive pulmonary disease and post-infectious sequelae as well as various autoimmune diseases. However, the single largest aetiology is idiopathic bronchiectasis, in which the underlying cause is unknown [1]. While each underlying aetiology considered individually may be rare, taken together, bronchiectasis occurs in up to 94.8 people per 100, 000 (0.1%) [2], increasing with age and female gender [3]. Prevalence is growing, and bronchiectasis has been described as an emerging global epidemic [4, 5].

Bronchiectasis is characterised by abnormal, scarred and irreversibly dilated bronchi [6]. The pathogenesis is not fully understood; however, once established, patients show evidence of chronic inflammation, infection, impaired mucociliary clearance and progressive structural lung damage. The complex interaction between these features (the so-called “vicious vortex”) leads to exacerbations and decline in pulmonary function, with associated morbidity and mortality [1].

Neutrophilic inflammation is a central feature of bronchiectasis, the extent of which has been associated with disease severity and progression [7, 8]. The release of inflammatory effectors from neutrophils contributes toward the inflammatory environment of the lung [9], and an imbalance between neutrophil-derived serine proteases (NSPs; neutrophil elastase [NE], proteinase 3 [PR3] and cathepsin G [CatG]) and their endogenous inhibitors has been documented in patients with chronic inflammatory respiratory diseases, including bronchiectasis [10–14]. This imbalance has been shown to impair defence against bacterial infection, impair mucociliary clearance, promote mucus hypersecretion and degrade elastin and other extracellular matrix components [10, 15]. NE has been shown to inhibit ciliary function and mucociliary clearance as well as promote mucus secretion in vitro, and NE activity in sputum is associated with disease severity, increased susceptibility to airway bacterial colonisation, increased risk/frequency of exacerbations, hospitalisations and mortality [16–19]. Levels of PR3 in sputum were found to be raised in patients with bronchiectasis during exacerbations compared with stable disease (i.e. not during an exacerbation) [20]. Additionally, PR3 concentration was correlated with levels of NE in sputum [20]. CatG has been implicated in the pathogenesis of bronchiectasis, resulting in destruction of airway epithelium and dysfunction of ciliated cells [21]. Increased CatG activity has been observed in patients with bronchiectasis compared with controls, with CatG activity increasing with increasing disease severity [21].

NE, PR3 and CatG are activated by cathepsin C (CatC; also known as dipeptidyl peptidase 1) during myelopoiesis of neutrophils in the bone marrow [22]. In the neutrophil progenitor cells, these NSPs are synthesised as pre-proenzymes that are trafficked to lysosomes, where they then are cleaved into proenzymes, finally activated by the removal of a prodipeptide by CatC into active mature enzymes and stored under acidic conditions (pH 4–5); non-activated NSPs are degraded prior to release of the mature neutrophils from the bone marrow [10, 22]. Inhibition of CatC in the bone marrow is therefore expected to result in circulating neutrophils with decreased levels of active NSPs recruited to the lungs of patients with chronic inflammatory lung diseases such as bronchiectasis. This, in turn, is expected to reduce distal airway destruction by enzymatic degradation of pulmonary elastin and connective tissue; possible secondary anti-inflammatory and anti-mucus hypersecretory effects are also expected [18, 23–25].

Currently available treatments for bronchiectasis, such as antibiotics, mucolytics and anti-inflammatories, are symptomatic only, with no drug licensed for treatment of the underlying pathophysiology, and the only evidence-based intervention to date is chest clearance [26, 27]. Further, in many cases, antibiotic treatment is insufficient to control infection, and even in the absence of infection, inflammation can progress [28]. Therefore, there is a high medical need for a novel treatment that breaks the vicious vortex of neutrophilic inflammation, prevents exacerbations and improves symptoms. Based on the aforementioned biology, inhibition of CatC is expected to have that potential. First evidence comes from the CatC inhibitor brensocatib (formerly AZD7986), which is currently in Phase 3 development for bronchiectasis (NCT04594369) [29]. BI 1291583 is a novel, highly potent and selective CatC inhibitor under investigation as a potential disease-modifying therapy for patients with bronchiectasis. In this study, we report the preclinical pharmacological in vitro and in vivo characterisation of BI 1291583.

## Methods

A brief summary of methods is given below. For detailed methodology, see Additional File 1. GraphPad Prism software (version 9.5.0 for Windows, GraphPad Software, www.graphpad.com) was used to generate all graphical representations of the data.

### Binding kinetics of BI 1291583 to human CatC

Binding kinetics of BI 1291583 to human CatC were assessed by surface plasmon resonance using a Biacore T200 system. Activated human CatC was immobilised on a CM5 chip using standard procedures. Increasing concentrations of BI 1291583 (0.08, 0.4, 2, 10 and 50 nM) were then injected onto the immobilised target at pH 4.5. Mean association rate constant (*k*_on_) and dissociation rate constant (*k*_off_) values were calculated from three individual experiments using Biacore T200 Evaluation Software, Version 1.0, 2010. The equilibrium dissociation constant (*K*_D_) was calculated from *k*_off_/*k*_on_, and half-life (*t*_1/2_) was calculated by ln_2_/*k*_off_.

### In vitro inhibition of recombinant human cathepsin enzymatic activity

Activity of recombinant human cathepsins in the presence of BI 1291583 was measured by conversion of fluorescent substrates for cathepsins C, K and L (duplicate experiments), and F, B, H and S (single experiments). Activated cathepsins were incubated with serial dilutions of BI 1291583 followed by addition of fluorescent cathepsin substrates. The reaction was stopped by addition of an inhibitor compound and fluorescence levels measured with an Envision Reader at excitation wavelength 355 nm, emission wavelength 460 nm, with vehicle controls as reference for non-inhibited enzyme activity and inhibitor as control for background fluorescence. Changes in fluorescence resulting from BI 1291583-mediated inhibition of cathepsins were determined by calculating the percentage of fluorescence in the presence of BI 1291583 compared with the fluorescence of the vehicle control after subtracting the background fluorescence. The concentration of BI 1291583 that inhibited 50% of cathepsin activity (IC_50_) was calculated using GraphPad Prism software (version 9.5.0 for Windows, GraphPad Software, www.graphpad.com) with a non-linear regression curve fit.

### Inhibition of the production of active NE in a neutrophil progenitor cell line

The inhibitory activity of BI 1291583 on the production of active NE was investigated in duplicate experiments in the human myeloid cell line U937. U937 cells were used as they constitutively express and process NE. Additionally, a myeloid cell line was used rather than primary neutrophils as CatC activates NSPs in the bone marrow during myelopoiesis and no further activation occurs once the neutrophils have entered the bloodstream; as such, the effect of CatC inhibition cannot be investigated in primary neutrophils. BI 1291583 was added to cell suspensions at a final concentration of 0.064, 0.32, 1.6, 8, 40, 200 and 1000 nM. Cells were then incubated for 48 h. Cell viability was then measured in duplicate experiments using the CellTiter-Glo^®^ Luminescent Cell Viability Assay according to manufacturer’s instructions. For measurement of NE activity, cells were separated by centrifugation, lysed and debris removed. NE activity was measured by conversion of a fluorescent substrate. Increase in fluorescence was measured over time with a SpectramaxM5 fluorescence reader at 360 nm excitation wavelength and 460 nm emission wavelength. The reaction rate when CatC is fully saturated by BI 1291583 was used as primary readout to calculate NE activity. Percentage inhibition of NE activity in lysates treated with BI 1291583 was calculated relative to the mean of the vehicle-only control. IC_50_ was calculated using GraphPad Prism software (version 9.5.0 for Windows, GraphPad Software, www.graphpad.com) with a non-linear regression curve fit.

The inhibitory effect of INS1007 (the active component of brensocatib) on the production of active NE was investigated under the same conditions (see Methods, Additional File 1).

### In vivo inhibition of the production of active NE, CatG and PR3

To demonstrate the ability of BI 1291583 to block the production of active NSPs, an in vivo proof-of-mechanism model was established in mice (female Crl:NMRI mice [*n* = 6 per treatment group]), and two dosing schedules were carried out. In the first—a 2-day treatment holiday regimen—mice were treated with 0.005, 0.05, 0.5 or 5 mg/kg BI 1291583, or vehicle, twice daily by oral gavage on Days 1‒5, 8 and 9. On Day 10, a final dose of BI 1291583 or vehicle was given followed by challenge with nebulised *E. coli* lipopolysaccharide (LPS) to induce neutrophil influx into the lungs. In the second dosing regimen, mice were treated with 0.00005, 0.0001, 0.001, 0.01, 0.03, 0.1, 0.5 or 5 mg/kg BI 1291583, or vehicle, once daily by oral gavage for 11 consecutive days. On Day 12, a final dose of BI 1291583 or vehicle was given followed by LPS challenge.

Four and a half hours after LPS challenge, lung neutrophils were harvested by lavage via tracheal cannula, and neutrophil count measured. Bronchoalveolar lavage fluid (BALF) samples were then centrifuged and cell pellets lysed with volume of lysis buffer normalised to neutrophil count. NE, CatG and PR3 activity were determined by conversion of a fluorescent substrate measured over time with a SpectramaxM5 fluorescence reader at 360 nm excitation wavelength and 460 nm emission wavelength. Fluorescence values were normalised to neutrophil count, and mean NE, CatG and PR3 activity from BI 1291583-treated animals was calculated relative to the mean of LPS/vehicle-treated control animals. CatG and PR3 activity was measured in pooled samples from each dose group. For the 11-day dosing experiments, the dose of BI 1291583 that inhibited 50% of NE activity (ED_50_) was calculated using GraphPad Prism software (version 9.5.0 for Windows, GraphPad Software, www.graphpad.com) with a non-linear regression curve fit. The dose inhibiting 99% of NE activity (ED_99_) was calculated from the regression curve using the equation ED_99_ = 99^(1/hill slope)^ × ED_50_. A one-way analysis of variance with Dunnett’s multiple comparisons test analysis was performed to determine statistical significance.

At 5 h after final BI 1291583 administration, animals were euthanised by Narcoren application (400–600 mg/kg intraperitoneally). For assessment of BI 1291583 exposure in plasma, five drops of retrobulbar blood were taken. The femur was prepared by extraction, shock frozen in liquid nitrogen and stored at − 20 °C until bone marrow preparation. For preparation of bone marrow, epiphysis severed off and bone marrow extracted from the diaphysis. Exposure was measured via liquid chromatography–tandem mass spectrometry.

The inhibitory activity of INS1007 on the production of active NE, and distribution of INS1007 to bone marrow and plasma was investigated under the same conditions as the 11-day dosing regimen (see Methods, Additional File 1).

### Selectivity analysis against unrelated proteases

Inhibitory and stimulatory activity of BI 1291583 (10 µM) against a panel of unrelated proteases from different classes was assessed by enzyme assays, using validated fluorometric or photometric techniques as appropriate. Effect was calculated as percentage inhibition (positive values) or stimulation (negative values) of a control inhibitor for each protease. An inhibition or stimulation of less than 25% was considered as insignificant, 25–50% was considered weak to moderate, and greater than 50% was considered significant. Experiments were carried out in duplicate and results presented as mean values.

## Results

### BI 1291583 binds human CatC in a reversible manner

BI 1291583 bound recombinant human CatC with mean *k*_on_ and *k*_off_ values at pH 4.5 of 6.36 × 10^6^ M^−1^ s^−1^ and 2.29 × 10^–3^ s^−1^, respectively, yielding a KD value of 0.43 nM. Mean *t*_1/2_ was 5.19 min. Kinetic parameters are summarised in Additional File 1: Table AF1.

### BI 1291583 selectively inhibits human CatC enzymatic activity in vitro

**Fig. 1:**
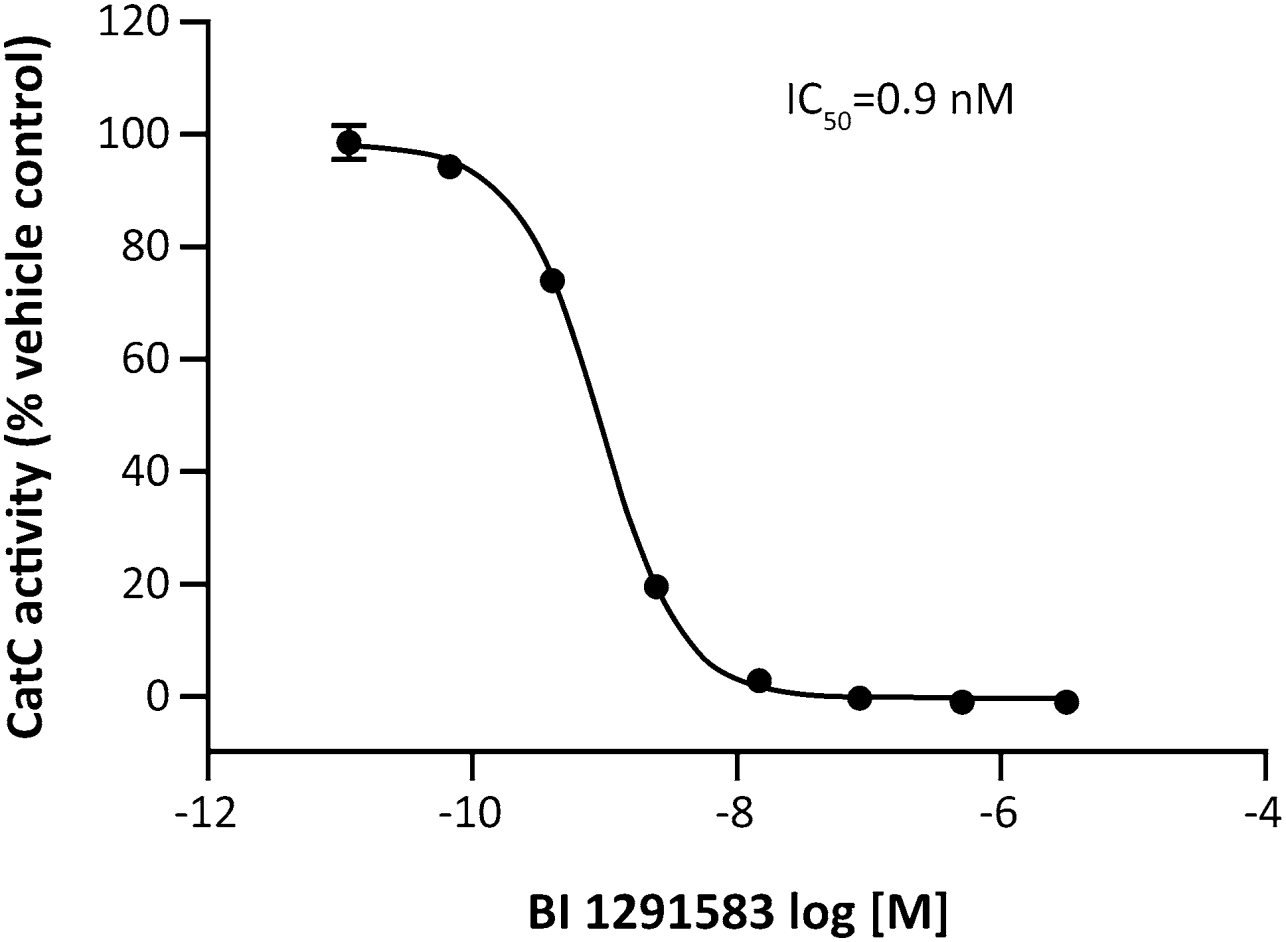
Inhibition of recombinant human CatC by BI 1291583. *CatC* cathepsin C, *IC*_*50*_ concentration of BI 1291583 resulting in 50% inhibition of cathepsin C enzymatic activity. Data are mean. Error bars depict standard deviation across duplicate experiments

Human CatC was inhibited with a mean IC_50_ of 0.9 nM (Fig. 1), with high selectivity among other related cysteine proteases (IC_50_ values ranging from 6695 nM for cathepsin K to greater than 100,000 nM for cathepsins B, F and H) (Table 1). Rat and mouse CatC were inhibited with similar potencies (mean IC_50_ values of 1.2 nM and 0.6 nM, respectively).

**Table 1:** Comparison of BI 1291583 enzyme selectivity for recombinant human cathepsins

Enzyme	IC_50_, nM
Cathepsin C	0.9
Cathepsin B	> 100,000
Cathepsin F	> 100,000
Cathepsin H	> 100,000
Cathepsin K	6695
Cathepsin L	7225
Cathepsin S	25,200

*IC*_*50*_ concentration of BI 1291583 resulting in 50% inhibition of cathepsin enzymatic activity

### BI 1291583 does not inhibit or stimulate other unrelated proteases

No relevant inhibition or stimulation of 33 unrelated proteases from four different classes was detected at 10 µM (Additional File 1: Table AF2).

### BI 1291583 inhibits the production of active NE in a neutrophil progenitor cell line

**Fig. 2:**
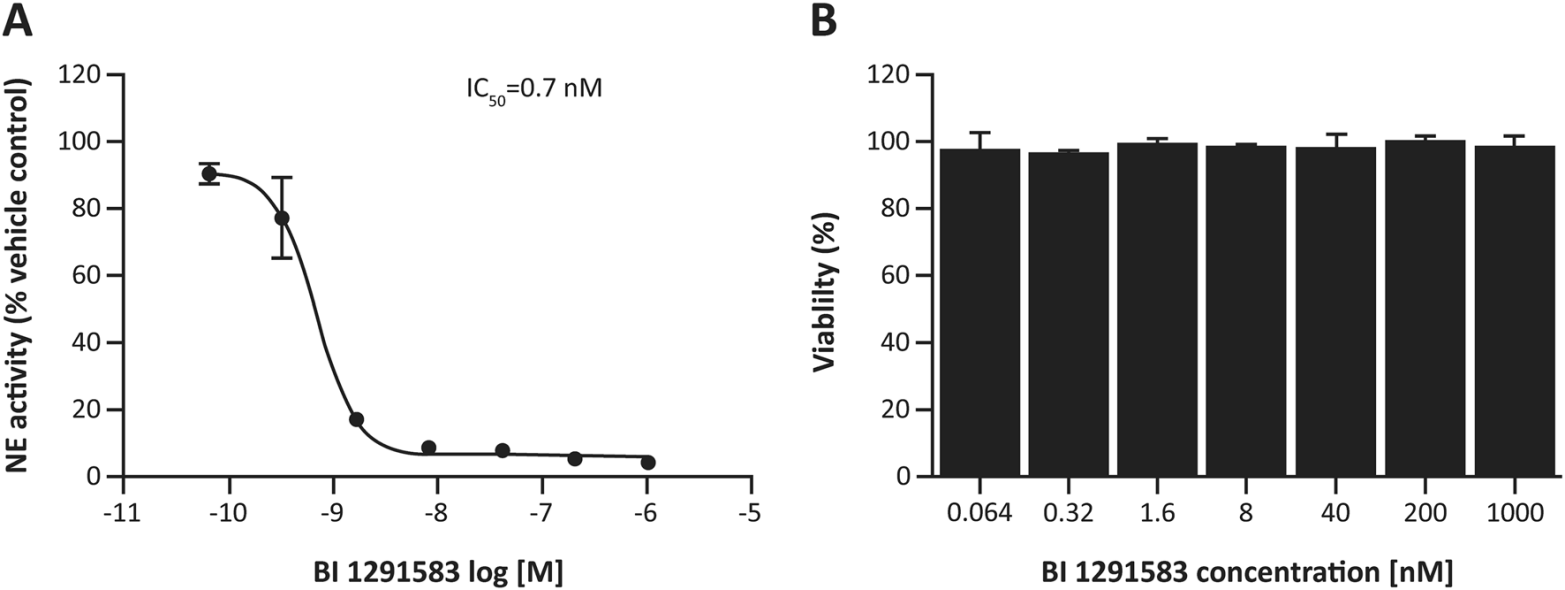
**A** Inhibition of the production of active NE in U937 cells by BI 1291583 and **B** effect on cell viability. *IC*_*50*_ concentration of BI 1291583 resulting in 50% inhibition of activation of neutrophil elastase, *NE* neutrophil elastase. Data are mean. Error bars depict standard deviation across duplicate experiments

BI 1291583 inhibited the production of active NE in neutrophil progenitor U937 cells in a concentration-dependent manner, with a mean IC_50_ of 0.7 nM, with no effects on cell viability (cell viability > 93% at all concentrations of BI 1291583 and a mean cell viability of 98% at the highest concentration [1000 nM]) (Fig. 2).

### BI 1291583 inhibits the production of active NE, PR3 and CatG in vivo

In vivo efficacy was evaluated in an LPS-challenge model in mice. In the 2-day treatment holiday regimen, BI 1291583 inhibited production of active NE in mouse BALF neutrophils in a dose-dependent manner up to a maximum of 97%. For NE analysis, BALF neutrophil lysates from animals treated with vehicle only and twice-daily dosing of 0.005, 0.05, 0.5 and 5 mg/kg BI 1291583 yielded mean (standard error of mean [SEM]) fluorescence signals of 2118.1 (132.3), 1008.3 (176.5), 458.1 (165.0), 81.1 (57.3) and 62.9 (46.8) relative fluorescence units (RFU) per 10^5^ neutrophils, respectively. These values translate to significant (*p* < 0.001) 44% (8.3%), 75% (7.8%), 96% (2.7%) and 97% (2.2%) inhibitions compared with vehicle-only treatment (Additional File Figure AF1, part A). An ED_99_ value for NE inhibition was calculated at 1.5 mg/kg. For CatG analysis, *V*_max_ values for vehicle only and 0.005, 0.05, 0.5 and 5 mg/kg BI 1291583 were 0.79, 0.28, 0.05, 0.01 and 0.01/10^5^ neutrophils, respectively, translating to 65%, 94%, 99% and 99% inhibitions compared with vehicle-only treatment (Additional File Figure AF1, part B).

**Fig. 3:**
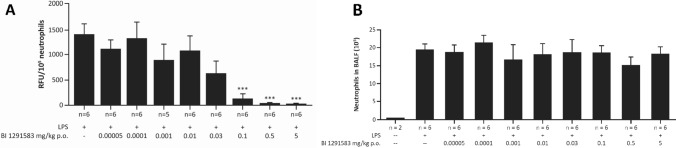
Effect of treatment with BI 1291583 on **A** NE activity in mouse BALF neutrophil lysate and **B** absolute neutrophil numbers in BALF after LPS challenge, 11-day dosing. *BALF* bronchoalveolar lavage fluid, *LPS* lipopolysaccharide, *NE* neutrophil elastase, *p.o.* orally, *RFU* relative fluorescence units. ***p < 0.001 compared with vehicle. Data are mean. Error bars indicate standard error of the mean

**Fig. 4:**
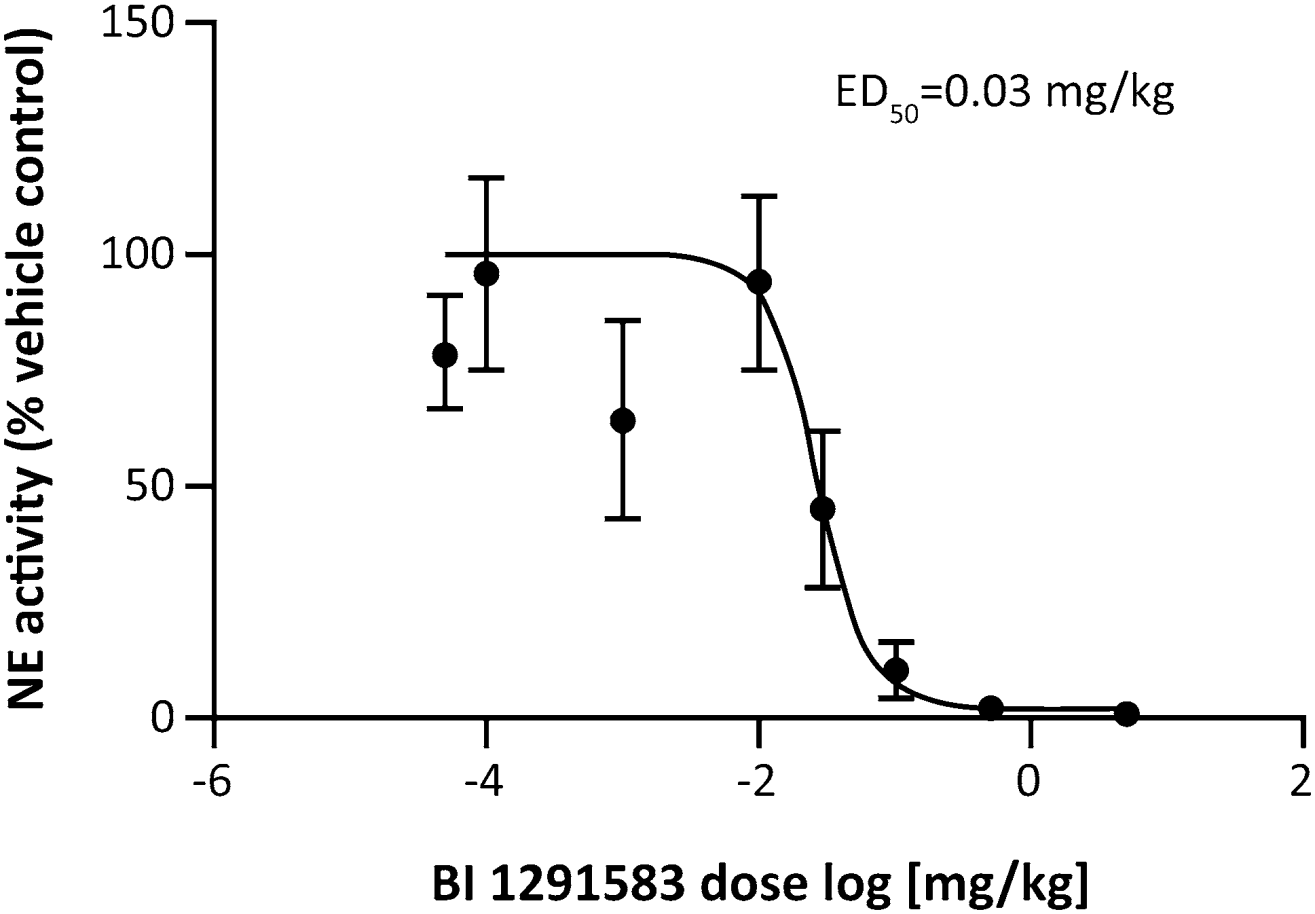
Dose–response for inhibition of active NE production in mouse BALF neutrophil lysate after treatment with BI 1291583 and subsequent LPS challenge, 11-day dosing. *ED*_*50*_ effective dose that inhibits 50% of production of active neutrophil elastase, *NE* neutrophil elastase. Data are mean. Error bars depict standard error of the mean

**Fig. 5:**
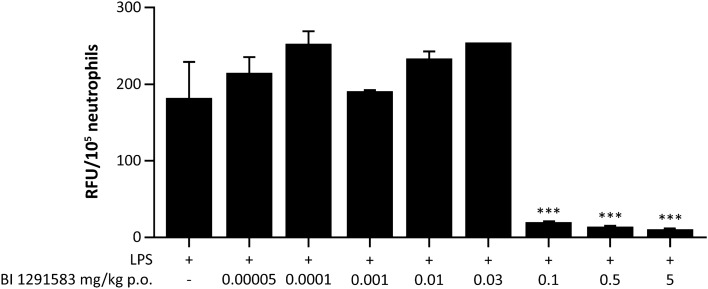
PR3 activity in mouse BALF neutrophil lysate after treatment with BI 1291583 and subsequent LPS challenge, 11-day dosing. *BALF* bronchoalveolar lavage fluid, *LPS* lipopolysaccharide, *p.o.* orally, *RFU* relative fluorescence units, *PR3* proteinase 3. ***p < 0.001 compared with vehicle. Samples were pooled for analysis of PR3 activity. Data are mean. Error bars depict standard error of the mean across duplicate experiments

Treatment with BI 1291583 for 11 days had no effect on LPS-induced neutrophil influx into the lung, but almost completely (99%) inhibited production of active NE in a dose-dependent manner. For NE analysis, BALF neutrophil lysates from animals treated with vehicle only and daily dosing of 0.1 mg/kg, 0.5 mg/kg and 5 mg/kg BI 1291583 yielded mean (SEM) fluorescence signals of 1419.2 (198.3), 147.8 (87.5), 27.0 (16.9) and 15.5 (4.6) relative fluorescence units (RFU) per 10^5^ neutrophils, respectively. These values translate to significant (*p* < 0.001) 90% (6.2%), 98% (1.2%) and 99% (0.3%) inhibitions compared with vehicle-only treatment (Fig. 3). The ED_50_ value for NE inhibition was calculated at 0.03 mg/kg, and ED_99_ was calculated at 0.3 mg/kg (Fig. 4). For PR3 analysis, values (SEM) for vehicle only and 0.1, 0.5 and 5 mg/kg BI 1291583 were 182 (47.5), 19 (0), 13.5 (1.5) and 10 (1.0) RFU/10^5^ neutrophils, respectively, translating to significant mean 90% (0%), 93% (0.8%) and 94% (0.6%) inhibitions compared with vehicle-only treatment (all *p* < 0.001) (Fig. 5).

### BI 1291583 distributes preferentially to bone marrow

At approximately 5 h after the final dose of BI 1291583 on Day 10 of 2-day treatment holiday regimen, mean (SEM) BI 1291583 exposure in bone marrow at 0.5 mg/kg was 2981.7 (185.0) nM. In plasma, the corresponding value was 32.5 (2.1) nM. This equates to a bone marrow-to-plasma exposure ratio of approximately 92 (Additional File Figure AF2).

**Fig. 6:**
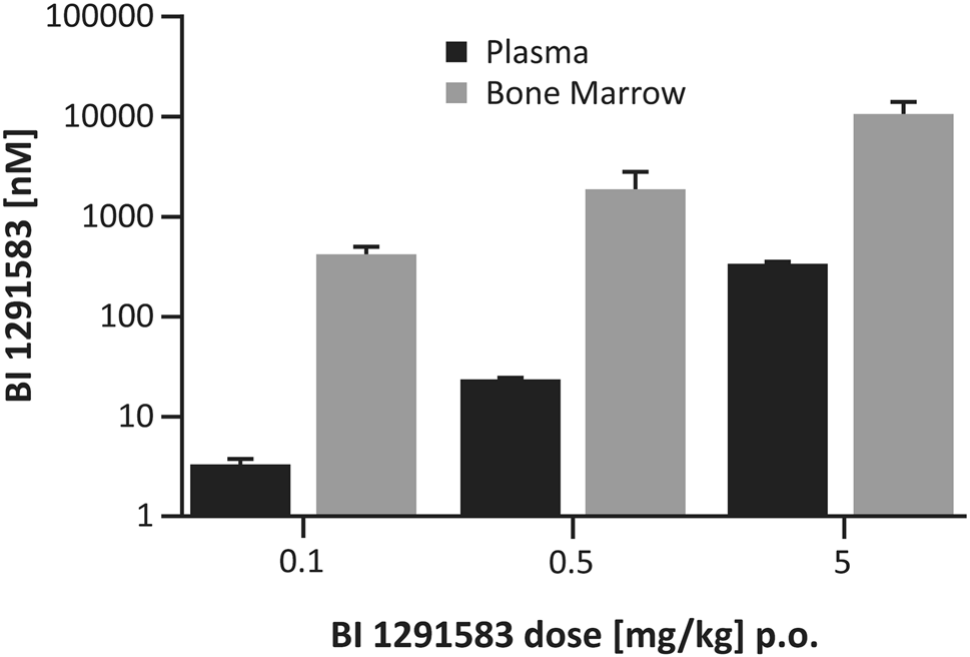
Bone marrow and plasma distribution of BI 1291583 at 5 h post-administration, 11-day dosing. *p.o.* orally. Data are mean. Error bars depict standard error of the mean

At 5 h after the final dose of BI 1291583 on Day 12 of the 11-day dosing regimen, mean (SEM) BI 1291583 exposure in bone marrow at efficacious doses (0.1, 0.5, 5 mg/kg) was 428.5 (83.6) nM, 1973.2 (908.9) nM and 11,092.0 (3287.5) nM, respectively. In plasma, corresponding values were 3.5 (0.4) nM, 24.2 (1.6) nM and 342.8 (24) nM. This equates to bone marrow-to-plasma exposure ratios of 122, 82 and 32 (Fig. 6).

The results of experiments investigating effects of INS1007 on production of active NE in U937 cells and the mouse LPS-challenge model, and analysis of distribution to bone marrow and plasma, can be found in Additional File 1.

## Discussion

In this comprehensive preclinical analysis, we demonstrated that BI 1291583 is a potent and selective inhibitor of CatC. Using SPR data, we could show that BI 1291583 binds human CatC in a reversible manner. It fully inhibited human recombinant CatC with a selectivity of greater than 6000-fold compared with a panel of related cathepsins, in addition to no relevant inhibition or stimulation of unrelated proteases from different classes. We also demonstrated similar potencies with mouse and rat CatC. We were then able to show that BI 1291583 had relevant downstream effects, dose-dependently inhibiting the production of active NE in a neutrophil progenitor cell line with no effect on cell viability. Finally, in the 2-day treatment holiday regimen, we demonstrated that BI 1291583 dose-dependently inhibited the production of active NE and CatG in BALF neutrophils by up to 97% and 99%, respectively. In the 11-day consecutive dosing model, we also demonstrated that BI 1291583 inhibited production of active NE and PR3 by up to 99% and 94%, respectively. At efficacious doses (those resulting in statistically significant inhibition of NSPs), BI 1291583 distributed to the target compartment of the bone marrow at up to 100 times the exposure compared with plasma, a property that can potentially be beneficial in reducing the risk of side effects due to the inhibition of CatC outside the bone marrow in patients.

There are few CatC inhibitors that have reached the level of clinical trials, and only two other compounds still in clinical development in bronchiectasis. HSK31858 is a CatC inhibitor that is currently being assessed in a Phase 2 clinical trial in patients with non-CF bronchiectasis (NCT05601778). Brensocatib (formerly AZD7986) is an oral, reversible CatC inhibitor currently in Phase 3 development for patients with bronchiectasis (NCT04594369). In Phase 1 development, healthy volunteers were randomised to once-daily doses of either brensocatib up to 40 mg or placebo for up to 28 days, and followed up for 1 month [30]. Exposure-related reduction in NE activity was demonstrated, with a generally well-tolerated safety profile; however, several dose-dependent, possibly CatC-related non-serious skin findings (exfoliation and hyperkeratosis) were observed [30]. In a subsequent Phase 2 trial [31], patients with bronchiectasis who had had at least two exacerbations in the previous year were randomised to once-daily doses of either 10 mg or 25 mg brensocatib or placebo for 24 weeks. Compared with placebo, at both brensocatib dose levels, the time to first exacerbation was significantly increased, risk of exacerbation was lower over the treatment period, and both annualised rates of exacerbations and the number of severe exacerbations were lower [31]. As in Phase 1 development, the incidence of hyperkeratosis was higher in the brensocatib-treated groups compared with the placebo group [31].

Brensocatib is at an advanced stage of clinical development. However, data presented here in the light of published data for brensocatib [32] suggest that BI 1291583 may also be a promising candidate for the treatment of patients with chronic inflammatory lung diseases, including bronchiectasis. We demonstrate that BI 1291583 inhibited recombinant human CatC with an IC_50_ of 0.9 nM; the published value for brensocatib is 4.0 nM [32]. Further, we have carried out an in-house analysis of INS1007—the active ingredient of brensocatib—under the same conditions as for BI 1291583, examining inhibition of NE in U937 cells, and NE inhibition and distribution to plasma and bone marrow in an in vivo mouse model (see Additional File 1). In this analysis, INS1007 inhibited the production of active NE in U937 cells with an IC_50_ of 64 nM; the value for BI 1291583 demonstrated in the study presented here is 0.7 nM. In the LPS challenge mouse model, INS1007 was tested at doses for which BI 1291583 resulted in statistically significant inhibition of NSPs for BI1291583 (0.1, 0.5 and 5 mg/kg). Overall, for INS1007, lower levels of inhibition of NE activation were observed compared with levels reported for BI 1291583 in this study (Additional File 1, Fig AF3). At the highest dose (5 mg/kg), INS1007 achieved up to 76% inhibition of NE activation in BALF neutrophils compared with vehicle control. BI1291583 shows 99% inhibition of NE activity at this dose (Additional File Figure AF4). At a low dose of 0.1 mg/kg, BI 1291583 resulted in 90% inhibition, whereas the same dose of INS1007 resulted in no inhibition, in line with the observed in vitro potencies. Analysis of INS1007 exposure at approximately 5 h after dosing at 0.1 mg/kg, 0.5 mg/kg and 5 mg/kg likewise revealed markedly lower bone marrow-to-plasma exposure ratios than those we report here for BI 1291583 (28, 4 and 2, respectively, for INS1007; 122, 81 and 32, respectively, for BI 1291583) (Additional File Figure AF5).

Overall, data from this in-house analysis of INS1007 strengthen the suggestion that BI 1291583 will be a promising candidate for the treatment of bronchiectasis, with a high bone marrow-to-plasma distribution ratio, high efficacy in both U937 cells and in vivo, and very low efficacious plasma concentrations, suggesting high target compartment efficacy.

In the present study, we demonstrate the bone marrow-to-plasma distribution profile of BI 1291583 in a mouse model. To investigate any interspecies differences in exposure, we carried out separate comparative kinetic studies in mice and rats after a single oral dose. In both species, we observed rapid distribution of BI 1291583 into bone marrow, with slow depletion (Additional File Figure AF6). Likewise, high bone marrow-to-plasma exposure ratios were observed, even at low doses, with no obvious difference between species. These data suggest that BI 1291583 has a rapid distribution into the target tissue in vivo, and complement the in vivo observations in both the 2-day treatment holiday regimen, and after 11 consecutive days of dosing in the present study.

A role for CatC in maintaining the structural integrity of plantar and palmar epidermal surfaces has been suggested, through processing of keratins in keratinocytes [33]. This suggestion is supported by the observation of dose-dependent skin events in the Phase 1 study of brensocatib [30]—the relative rapidity of onset of these events did not correlate with the dynamics of NE activity. Further, in the Phase 1 study of the irreversible CatC inhibitor GSK2793660 [34], marked skin desquamation events were observed along with insufficient inhibition of downstream NSPs, which led to trial termination. We suggest that the preferential distribution of BI 1291583 to the bone marrow observed in our study may minimise skin events in subsequent clinical trials.

Increased levels of sputum PR3 have been previously demonstrated alongside NE in patients with bronchiectasis during exacerbations [20]; similarly, CatG has also been implicated in the pathogenesis of bronchiectasis alongside NE [21]. Thus, our finding of a dose-dependent significant decrease in production of active PR3 and CatG in vivo by BI 1291583 suggests that upstream inhibition of CatC, and the resulting broad inhibition of NSPs, may add further beneficial therapeutic potential in patients with bronchiectasis.

## Conclusion

This study demonstrates that BI 1291583 is a reversible, highly potent and highly selective inhibitor of CatC, which has the potential to ameliorate neutrophilic inflammation and tissue destruction mediated by uncontrolled NSP activity in the airways. Results of this preclinical study support further clinical investigation of BI 1291583 in patients with bronchiectasis.

## Supplementary Information

Below is the link to the electronic supplementary material.

Supplementary file1 (DOCX 721 KB)

## Data Availability

The datasets used and/or analysed during the current study are available from the corresponding author on reasonable request.

## Abbreviations

BALF: Bronchoalveolar lavage fluid
CatC: Cathepsin C
CatG: Cathepsin G
ED_50_: Effective dose of drug that inhibits 50% of target activity
ED_99_: Effective dose of drug that inhibits 99% of target activity
IC_50_: Concentration of drug resulting in 50% inhibition of target
K_D_: Equilibrium dissociation constant
*k*_on_: Mean association rate constant
*k*_off_: Mean dissociation rate constant
LPS: Lipopolysaccharide
NE: Neutrophil elastase
NSP: Neutrophil-derived serine protease
PR3: Proteinase 3
RFU: Relative fluorescence units
SEM: Standard error of mean
*t*_1/2_: Half-life (*t*_1/2_)
