# Supplementary material for: BI 1291583: a novel selective inhibitor of cathepsin C with superior in vivo profile for the treatment of bronchiectasis
Source: Inflamm Res. 2023 Aug 4;72(8):1709–17. doi: 10.1007/s00011-023-01774-4 (PMC10499737; doi:10.1007/s00011-023-01774-4)
Supplement: Supplementary file 1 — Supplementary file1 (DOCX 721 KB) [file 11_2023_1774_MOESM1_ESM.docx]

**Additional file 1**

**BI 1291583: a novel selective inhibitor of cathepsin C with superior *in vivo* profile for the treatment of bronchiectasis**

Stefan Kreideweiss^1^, Gerhard Schänzle^1^, Gisela Schnapp^1^, Viktor Vintonyak^1^, Marc A. Grundl^1^

^1^Boehringer Ingelheim Pharma GmbH & Co. KG, Biberach, Germany

**Methods**

*Binding kinetics of BI 1291583 to human cathepsin C*

Preparation of test compound

BI1291583 was dissolved to a 20 mM stock solution in DMSO. This stock solution was initially diluted 100-fold in running buffer (10 mM HEPES pH 7.4, 150 mM NaCl) to a concentration of 200 µM and 1% DMSO. The 200 µM BI1291583 stock solution in 1% DMSO was then further diluted serially 1:5 in running buffer + 1% DMSO. Accordingly, the final compound concentrations in the assay were 0.05, 0.01, 0.002, 0.0004 and 0.00008 µM) in running buffer with 1% DMSO. All interaction experiments were performed at 25°C in 10 mM Na-acetate pH 4.5; 150 mM NaCl; 1 mM TCEP; 0.05% Tween; 1% DMSO.

Surface plasmon resonance

CatC was immobilised by amine coupling on a CM5 chip using standard procedures. The protein was activated with 1 mM cysteamine for 10 min at 4°C, before CatC was pre-incubated with a reversible active site binder to protect the active site and diluted to around 0.04 mg/ml using 10 mM Na-acetate pH 5.0 prior to immobilisation. The immobilisation level was in the range of 8000–10000 RU.

All interaction experiments were performed at 25°C in 10 mM Na-acetate pH 4.5; 150 mM NaCl;
1 mM TCEP; 0.05% Tween; 1% DMSO. The compound was diluted in the running buffer and injected at a flow rate of 30 µl/min for 180 s via the immobilised target protein at increasing concentrations (5 concentrations between 0.05 µM and 0.00008 µM in a single cycle kinetic experiment). Dissociation time was 3600 s.

Sensorgrams from reference surfaces and blank injections were subtracted from the raw data prior to data analysis, using Biacore T200 evaluation software 1.0. A 1:1 interaction model, with a term for mass-transport included, was fitted globally to sensorgrams recorded at different compound concentrations in single-cycle experiments.

In vitro *inhibition of recombinant human cathepsin enzymatic activity*

Preparation of test compound

**CatC**
BI 1291583 was dissolved in DMSO at a concentration of 10 mM and further diluted in DMSO to obtain a 300 µM stock solution. Afterwards, seven 1:6 dilution steps in DMSO were performed followed by a 1:25 dilution step in ddH_2_O of each of these solutions. 5 µl of these final dilutions were used in the experiments resulting in a series of assays containing 3000 nM, 500 nM, 83 nM, 14 nM, 2.3 nM, 0.39 nM, 0.064 nM and 0.011 nM BI 1291583 final concentrations.

**CatK and CatL**BI 1291583 was dissolved in DMSO at a concentration of 10 mM and further diluted in DMSO to obtain a 3000 µM stock solution. Afterwards, seven 1:6 dilution steps in DMSO were performed followed by a 1:25 dilution step in ddH_2_O of each of these solutions. 5µl of these final dilutions were used in the experiments resulting in a series of assays containing 30000 nM, 5000 nM, 830 nM, 140 nM, 23 nM, 3.9 nM, 0.64 nM and 0.11 nM BI 1291583 final concentrations.

**CatB, CatF, CatH and CatS**BI 1291583 was dissolved in DMSO at a concentration of 10 mM. Afterwards, seven 1:5 dilution steps in DMSO were performed followed by a 1:25 dilution step in ddH_2_O of each of these solutions. 5 µl of these final dilutions were used in the experiments resulting in a series of assays containing 100 µM, 20 µM, 4 µM, 800 nM, 160 nM, 32 nM, 6.4 nM and 1.8 nM BI 1291583 final concentrations.

Activation of cathepsins

**CatC**Recombinant human CatC (Prozymex ZU-24) was diluted to 35 µg/ml in TAGzyme buffer (20 mM NaH_2_PO_4_ pH 6.0, 150 mM NaCl). 50 µl of this solution was mixed with 100 µl 2 mM cysteamine and incubated for 5 min at room temperature. After the incubation, the activated CatC was diluted to 0.025 ng/µl in MES buffer (25 mM MES pH 6.0, 50 mM NaCl, 5 mM DTT, 0.1% BSA) and used for the CatC assay.

**CatK**32.5mM NaOAc, pH 3.5 was added to 20% of recombinant human pro-CatK (Proenzyme; Enzo Life Science #BML-SE367-0050) volume for a final working concentration of 300 ng/µl and incubated for 30 min at room temperature.

**CatL**Recombinant human CatL (R&D systems #952-CY) was diluted to 40 µg/ml in assay buffer (400 mM NaOAc pH 5.5, 4 mM EDTA, 0.003% Brji35, 8 mM DTT) and incubated for 15 min on ice. It was further diluted in assay buffer to obtain a 0.0025 ng/µl working solution.

**CatF**1 µl of recombinant human CatF (Enzo Life Sciences # BML-SE541; 58 ng/µl) was mixed with 1 µl activation buffer (100mM NaOAc pH4.2, 2.5mM EDTA, 0.01% Brij 35, 0.8mg/ml Pepsin (Sigma Aldrich #B4184), 5mM DTT), incubated for 1h at 37°C and diluted to 0.125 ng/µl in assay buffer (50mM KH_2_PO_4_, 2.5mM EDTA, 0.01% Brij 35, 2.5mM DTT) to obtain the final working solution.

**CatB**

A working 0.05 ng/μl solution of recombinant human CatB (Enzo Life Sciences #BML-SE198) was prepared in assay buffer (50mM KH_2_PO_4_ pH 6.5, 50mM NaCl, 2mM EDTA, 0.5mM DTT, 0.1% BSA).

**CatH**

A working 2 ng/μl solution of recombinant human CatH (Enzo Life Sciences #BML-SE200) was prepared in assay buffer (200mM KH_2_PO_4_ pH 6.8, 4mM EDTA, 0.1% BSA, 0.01% Brji35, 8 mM DTT).

**CatS**

A working 0.025 ng/μl solution of recombinant human CatS (Enzo Life Sciences #BML-SE453) was prepared in assay buffer (50 mM KH_2_PO_4_ pH 6.5, 50 mM NaCl, 2 mM EDTA, 0.1% BSA, 0.01% Triton X-100, 0.5 mM DTT).

Activity assays

**CatC**CatC activity was measured by the conversion of the fluorescent substrate Gly-Arg-AMC (Biotrend # 808756). The substrate was prepared as 100 mM stock solution in DMSO and diluted in MES buffer (25 mM MES pH 6.0, 50 mM NaCl, 5 mM DTT, 0.1% BSA) to obtain a 200 µM working solution.

To stop the CatC reaction at the end of the assay, the inhibitor Gly-Phe-DMK (MP Biomedicals #03DK00625) was prepared as 100 mM stock solution in DMSO, diluted in DMSO to 10 mM and finally diluted in MES buffer to obtain a 10 µM working solution.

In 384-well plates, 5 µl test compound dilution were mixed with 10 µl of CatC in MES buffer (final enzyme concentration 0.0125 ng/µl) followed by a 10-min incubation at room temperature. Then 5 µl CatC substrate in MES buffer (final concentration 50 µM) were added. The plates were then incubated at room temperature for 30 min. The reaction was stopped with 10 µl of Gly-Phe-DMK in MES-buffer (final concentration 3.3 µM). The fluorescence of the wells was determined with an Envision Reader (PerkinElmer; excitation wavelength 355 nm, emission wavelength 460 nm).

Each microtitre plate contained wells with vehicle controls (1% DMSO in ddH_2_O + 0.075% BSA) as reference for non-inhibited enzyme activity (100% control; high values) and wells with inhibitor (Gly-Phe-DMK in ddH_2_O + 1% DMSO + 0.075% BSA, final concentration 1µM) as controls for background fluorescence (0% control; low values).

Data analysis was performed by the calculation of the percentage of fluorescence in the presence of the test compound compared to the fluorescence of the vehicle control after subtracting the background fluorescence: (RFU(sample)-RFU(background))*100/(RFU(control)-RFU(background))

IC_50_ was calculated using GraphPad Prism software (version 9.5.0 for Windows, GraphPad Software, www.graphpad.com) with a non-linear regression curve fit. The IC_50_ value was interpolated as the concentration of test compound which inhibits 50% of CatC activity.

**CatK**CatK activity was measured by the conversion of the fluorescent substrate Z-GPR-AMC (Enzo Life Sciences #BML-P142). The substrate was prepared as 20 mM stock solution in DMSO and diluted in assay buffer (150 mM NaOAc pH 5.5, 4 mM EDTA, 20 mM L-cysteine) to obtain a 50 µM working solution.

To stop the CatK reaction at the end of the assay, the protease inhibitor E64 (SigmaAldrich #E3132) was prepared as 10 mM stock solution in DMSO and diluted in assay buffer to obtain a 3 µM working solution.

In 384-well plates, 5 µl test compound were mixed with 10 µl of enzyme in assay buffer (final enzyme concentration 2 ng/µl) followed by 10-min incubation at room temperature. Then 5 µl substrate in assay buffer (final concentration 12.5 µM) were added followed by 60 min incubation at room temperature. The reaction was stopped with 10 µl of E64 in assay buffer (final concentration 1µM). The fluorescence in the wells was measured using an Envision reader (PerkinElmer; excitation wavelength 360nm, emission wavelength 460 nm).

The microtitre plate contained wells with vehicle controls (1% DMSO in ddH_2_O) as reference for non-inhibited enzyme activity (100% control; high values) and wells with inhibitor (E64 in ddH_2_O + 1% DMSO, final concentration 1 µM) as controls for background fluorescence (0% control; low values).

Data analysis and calculation of IC_50_ was carried out as above.

**CatL**
CatL activity was measured by the conversion of the fluorescent substrate Z-Phe-Arg-AMC (Bachem #I-1160.0050). The substrate was prepared as 10 mM stock solution in DMSO and diluted in assay buffer (400 mM NaOAc pH 5.5, 4 mM EDTA, 0.003% Brji35, 8 mM DTT) to obtain a 40 µM working solution.

To stop the CatL reaction at the end of the assay, the protease inhibitor NapSul-Ile-Trp-CHO (Enzo Life Sciences #BML-PI125) was prepared as 10 mM stock solution in DMSO and diluted in assay buffer to obtain a 30 µM working solution.

In 384-well plates, 5 µl test compound dilution were mixed with 10 µl of enzyme in assay buffer (final enzyme concentration 0.00125 ng/μl) followed by a 10-min incubation at room temperature. Then 5 µl substrate in assay buffer (final concentration 10 µM) were added. The plates were then incubated at room temperature for 30 min. The reaction was stopped by adding 10 µl NapSul-Ile-Trp-CHO in assay buffer (final concentration 10 µM). The fluorescence in the wells was determined with an Envision reader (Perkin Elmer; excitation wavelength 355 nm, emission wavelength 460 nm).

Each assay microtitre plate contained wells with vehicle controls (1% DMSO + 0.075% BSA in ddH_2_O) as reference for non-inhibited enzyme activity (100% control; high values) and wells with inhibitor (NapSul-Ile-Trp-CHO in ddH_2_O + 1% DMSO + 0.075% BSA, final concentration 10 µM) as controls for background fluorescence (0% control; low values).

Data analysis and calculation of IC_50_ was carried out as above.

**CatF**
CatF activity was measured by the conversion of the fluorescent substrate Z-Leu-Arg-AMC (Enzo Life Sciences #BML-P229). The substrate was prepared as 50 mM stock solution in DMSO and diluted in assay buffer (50 mM KH_2_PO_4_, 2.5 mM EDTA, 0.01% Brij 35, 2.5 mM DTT) to obtain a 40 µM working solution.

To stop the CatF reaction at the end of the assay, the protease inhibitor E64 (SigmaAldrich #E3132) was prepared as 10 mM stock solution in DMSO and diluted in assay buffer to obtain a 3 µM working solution.

In 384-well plates, 5 µl test compound dilution were mixed with 10 µl of enzyme in assay buffer (50 mM KH_2_PO_4_, 2.5mM EDTA, 0.01% Brij 35, 2.5 mM DTT (final enzyme concentration 0.0625 ng/µl) followed by a 10-min incubation at room temperature. Then 5 µl substrate in assay buffer (final concentration 10 µM) were added. The plates were then incubated at room temperature for 30 min. The reactions were stopped with 10 µl of the protease inhibitor E64 in assay buffer (final concentration 10 µM). The fluorescence in the wells was determined with a Molecular Devices SpectraMax M5 Reader (excitation wavelength 360 nm, emission wavelength 460 nm).

Each microtitre plate contained wells with vehicle controls (1% DMSO in ddH_2_O) as reference for non-inhibited enzyme activity (100% control; high values) and wells with inhibitor (E64 in ddH_2_O + 1% DMSO, final concentration 10 µM) as controls for background fluorescence (0% control; low values).

Data analysis and calculation of IC_50_ was carried out as above.

**CatB**
CatB activity was measured by the conversion of the fluorescent substrate Z-Arg-Arg-AMC (Enzo Life Sciences #BML-P137). The substrate was prepared as 80 mM stock solution in DMSO and diluted in assay buffer (50 mM KH_2_PO_4_ pH 6.5, 50 mM NaCl, 2 mM EDTA, 0.5 mM DTT, 0.1% BSA) to obtain a 400 µM working solution.

To stop the CatB reaction at the end of the assay, the protease inhibitor CA-074 (Enzo Life Sciences #BML-PI131) was prepared as 10 mM stock solution in DMSO and diluted in assay buffer to obtain a 30 µM working solution.

In 384-well plates, 5 µl test compound dilution were mixed with 10 µl of enzyme in assay buffer (final enzyme concentration 0.025 ng/µl) followed by a 10-min incubation at room temperature. Then 5 µl substrate in assay buffer (final concentration 100 µM) were added. The plates were then incubated at room temperature for 30 min. The reaction was stopped by adding 10 µl CA-074 in assay buffer (final concentration 10µM). The fluorescence in the wells was determined with a Molecular Devices SpectraMax M5 Reader (excitation wavelength 360 nm, emission wavelength 460 nm).

Each assay microtitre plate contained wells with vehicle controls (1% DMSO + 0.075% BSA in ddH_2_O) as reference for non-inhibited enzyme activity (100% control; high values) and wells with inhibitor (CA-074 in ddH_2_O + 1% DMSO + 0.075% BSA, final concentration 10 µM) as controls for background fluorescence (0% control; low values).

Data analysis and calculation of IC_50_ was carried out as above.

**CatH**
CatH activity was measured by the conversion of the fluorescent substrate H-Arg-AMC (Enzo Life Sciences #BML-P135). The substrate was prepared as 100 mM stock solution in DMSO and diluted in assay buffer (200 mM KH_2_PO_4_ pH 6.8, 4 mM EDTA, 0.1% BSA, 0.01% Brji35, 8 mM DTT) to obtain a 200 µM working solution.

To stop the CatH reaction at the end of the assay, the protease inhibitor E64 (SigmaAldrich #E3132) was prepared as 10 mM stock solution in DMSO and diluted in assay buffer to obtain a 30 µM working solution.

In 384-well plates, 5 µl test compound dilution were mixed with 10 µl of enzyme in assay buffer (final enzyme concentration 1 ng/μl) followed by a 10-min incubation at room temperature. Then 5 µl substrate in assay buffer (final concentration 50 µM) were added. The plates were then incubated at room temperature for 60 min. The reaction was stopped by adding 10 µl E64 in assay buffer (final concentration 10 µM). The fluorescence in the wells was determined with a Molecular Devices SpectraMax M5 Reader (excitation wavelength 360 nm, emission wavelength 460 nm).

Each assay microtitre plate contained wells with vehicle controls (1% DMSO + 0.075% BSA in ddH_2_O) as reference for non-inhibited enzyme activity (100% control; high values) and wells with inhibitor (CA-074 in ddH_2_O + 1% DMSO + 0.075% BSA, final concentration 10 µM) as controls for background fluorescence (0% control; low values).

Data analysis and calculation of IC_50_ was carried out as above.

**CatS**
CatS activity was measured by the conversion of the fluorescent substrate Z-Val-Val-Arg-AMC (Enzo Life Sciences #BML-P199. The substrate was prepared as 10 mM stock solution in DMSO and diluted in assay buffer (50 mM KH_2_PO_4_ pH 6.5, 50 mM NaCl, 2 mM EDTA, 0.1% BSA, 0.01% Triton X-100, 0.5 mM DTT) to obtain a 40 µM working solution.

To stop the CatS reaction at the end of the assay, the protease inhibitor Z-Phe-Leu-COCHO (Calbiochem #219393) was prepared as 10 mM stock solution in DMSO and diluted in assay buffer to obtain the 3 µM working solution.

In 384-well plates, 5 µl test compound dilution were mixed with 10 µl of enzyme in assay buffer (final enzyme concentration 0.0125 ng/µl) followed by a 10-min incubation at room temperature. Then 5 µl substrate in assay buffer (final concentration 10 µM) were added. The plates were then incubated at room temperature for 30 min. The reaction was stopped by adding 10 µl Z-Phe-Leu-COCHO in assay buffer (final concentration 1 µM). The fluorescence in the wells was determined with a Molecular Devices SpectraMax M5 Reader (excitation wavelength 360 nm, emission wavelength 460 nm).

Each assay microtitre plate contained wells with vehicle controls (1% DMSO + 0.075% BSA in ddH_2_O) as reference for non-inhibited enzyme activity (100% control; high values) and wells with inhibitor (Z-Phe-Leu-COCHO in ddH_2_O + 1% DMSO + 0.075% BSA, final concentration 10 µM) as controls for background fluorescence (0% control; low values).

Data analysis and calculation of IC_50_ was carried out as above.

In vitro *inhibition of recombinant mouse CatC enzymatic activity*

Preparation of test compound

BI 1291583 was dissolved in DMSO at a concentration of 10 mM and further diluted in DMSO to obtain a 10 µM stock solution. Afterwards, seven 1:5 dilution steps in DMSO were performed, followed by a 1:25 dilution step in ddH_2_O of each of these solutions. 5 µl of these final dilutions were used in the experiments, resulting in a series of assays containing 100 nM, 20 nM, 4 nM, 0.8 nM, 0.16 nM, 0.032 nM, 0.0064 nM and 0.00128 nM BI 1291583 final concentrations.

Activation

Recombinant mouse CatC (R&D Systems #2336-CY) was diluted in MES buffer (25 mM MES pH 6.0, 50 mM NaCl, 5 mM DTT, 0.1% BSA) to obtain a 25 μg/µl working solution. Activation was as described above for measurement of human CatC activity.

Activity assay

CatC activity was measured by the conversion of the fluorescent substrate Gly-Arg-AMC (Biotrend # 808756). The substrate was prepared as 100 mM stock solution in DMSO and diluted in MES buffer (25 mM MES pH 6.0, 50 mM NaCl, 5 mM DTT, 0.1% BSA) to obtain a 200 µM working solution.

To stop the CatC reaction at the end of the assay, the inhibitor Gly-Phe-DMK (MP Biomedicals #03DK00625) was prepared as 100 mM stock solution in DMSO, diluted in DMSO to 10 mM and finally diluted in MES buffer to obtain a 3 µM working solution.

In 384-well plates, 5 µl of test compound dilution were mixed with 10 µl of CatC in MES buffer (final enzyme concentration 0.0125 ng/µl) followed by a 10-min incubation at room temperature. Then 5 µl of CatC substrate in MES buffer (final concentration 50 µM) were added. The plates were then incubated at room temperature for 30 min. The reaction was stopped with 10 µl of Gly-Phe-DMK in MES buffer (final concentration 1 µM). The fluorescence of the wells was determined with a Molecular Devices SpectraMax M5 Reader (excitation wavelength 360 nm, emission wavelength 460 nm).

Microtitre plate set-up for vehicle and inhibitor controls, and data analysis were as per measurement of human CatC activity above.

In vitro *inhibition of recombinant rat CatC enzymatic activity*

Preparation of test compound

*Experiment 1*

BI 1291583 was dissolved in DMSO, and 1:5 and 1:25 dilutions in ddH_2_O were carried out as per measurement of mouse CatC activity above. 5 µl of these final dilutions were used in the experiments, resulting in a series of assays containing 1000 nM, 200 nM, 40 nM, 8 nM, 1.6 nM, 0.32 nM, 0.064 nM and 0.0128 nM BI 1291583 final concentrations.

*Experiment 2*

Final concentrations of BI 1291583 were prepared as per Experiment 1, but diluted to obtain 100 nM, 20 nM, 4 nM, 0.8 nM, 0.16 nM, 0.032 nM, 0.0064 nM and 0.00128 nM BI 1291583 final concentrations.

Activation

Recombinant rat CatC (Prozymex UZ-24) was diluted in TAGzyme buffer (20 mM NaH_2_PO_4_ pH 6.0, 150 mM NaCl) to obtain a 38.1 µg/µl working solution. Activation was as described above for measurement of human CatC activity.

Activity assay

Activity of rat CatC in Experiments 1 and 2 was measured as per measurement of mouse CatC activity above.

*Inhibition of the production of active NE by BI 1291583 in a neutrophil progenitor cell line*

Preparation of test compound

BI 1291583 was dissolved in DMSO at a concentration of 1 mM in DMSO and diluted 1:10 in RPMI 1640 medium (Gibco # 68170). Seven 1:5 dilution steps in RPMI 1640 medium (+ 10% DMSO) were performed, and 20 µl per well of these dilutions were added to 2 ml cell suspension (human myeloid cell line U937), resulting a series of assays containing 1 µM, 0.2 µM, 40 nM, 8 nM, 1.6 nM, 0.32 nM and 0.064 nM BI 1291583.

Incubation of U937 cells with BI 1291583

2 ml cell suspensions (10^5^ cells/ml in RPMI + 10% FCS + 25 mM HEPES) were mixed with 20 µl of the respective compound dilution in a 24 well plate (FisherScientific #10604903; two wells per compound concentration). Control wells contained 2 ml cell suspension + 0.1% DMSO (final concentration). The cells were incubated for 48h at 37°C, 5% CO_2_, 96% relative humidity.

Measurement of cell viability

100 µl cell suspension were transferred into a white 96 well plate (ThermoFisherScientific #10072151) and cell viability determined using the “CellTiter-Glo® Luminescent Cell Viability Assay” (Promega # G7570) according to the supplier’s protocol. The mean luciferase activity in the control wells was set at 100% and the luciferase activity in the compound-treated cells was calculated relative to this value.

Preparation of cell lysates and measurement of neutrophil elastase activity

Cells were separated by centrifugation (10 min, 500g, room temperature) and washed twice with 1 ml PBS (Lonza #BE17-516F) each. Cells were separated by centrifugation as above, supernatant was removed and the cell pellet in each well was resuspended into 80 µl lysis buffer (20 mM Tris-HCl pH 7.5, 100 mM NaCl, 0.2% NP40) and transferred into a 96-well plate (Greiner #651101). The plates were incubated for 10 min on ice, cell debris was removed by centrifugation (10 min, 1000g, 4°C) and the lysate transferred into a new 96-well plate. Cell lysates were stored at -20°C until further analysis. Total protein concentration in the lysates was determined using a Pierce™ BCA Protein Assay Kit (ThermoFisherScientific #23225) according to the supplier’s protocol.

Neutrophil elastase (NE) activity was measured by the conversion of the fluorescent substrate MeOSuc-Ala-Ala-Pro-Val-AMC (Bachem #I-1270) in the cell lysates. The substrate was prepared as 1 mM stock solution in Tris-buffer (100mM Tris; 1M NaCl; pH 7.5). Cell lysates were diluted to 0.5 mg/ml total protein in lysis buffer (+0.1% HSA) for the NE measurement.

A NE standard curve was prepared by using a 100 µg/ml stock solution of human NE (Calbiochem #324681) in Tris-buffer (100 mM Tris; 1M NaCl; pH 7.5). This was further diluted in Tris-buffer with 0.1% HSA (Calbiochem #126658) to 750 ng/ml and further serial diluted in 1:2 steps in Tris-buffer (+0.1% HSA) for the final standard curve.

Cell lysates, blank controls and the NE standard curve were mixed in a 384-well plate as follows:

- Blank: 5 µl Tris buffer + 10 µl Tris buffer (+ 0.1% HSA) + 5 µl NE substrate (1 mM)
- Standard curve: 5 µl Tris buffer + 10 µl NE dilution + 5 µl NE substrate (1 mM)
- 5 µl Tris buffer + 10 µl cell lysate + 5 µl NE substrate (1 mM)

The increase in fluorescence was measured over 30 min with a fluorescence reader (SpectramaxM5, Molecular devices) with 360 nm excitation wavelength and 460 nm emission wavelength. V_max_(units/sec) was used as primary read-out to calculate NE activity. The amount of neutrophil elastase (ng/ml) was calculated using the standard curve and interpolated to ng/mg total lysate protein. Percent inhibition of NE activity in the compound-treated lysate samples is calculated relative to the mean of the DMSO-treated control-sample:
(100-[NE(compound)*100/NE(mean_control)]).

IC_50_ was calculated using GraphPad Prism software (version 9.5.0 for Windows, GraphPad Software, www.graphpad.com) with a non-linear regression curve fit. The IC_50_ value was interpolated as the concentration of test compound which inhibits 50% of neutrophil elastase activity (relative to the DMSO-treated control).

*Inhibition of the production of active NE by INS1007 in a neutrophil progenitor cell line*

Preparation of INS1007, incubation with U937 cells, preparation of cell lysates and measurement of NE activity were as described for BI 1291583.

In vivo *inhibition of NE, CatG and PR3 activity*

Preparation of test compound

BI 1291583 was dissolved in 1M HCl and mixed with 0.5% Natrosol to obtain a 0.5 mg/ml application solution. This solution was further diluted in vehicle solution (2.5 ml 1 M HCl + 300 ml 0.5% Natrosol [hydroxyethylcellulose; SigmaAldrich #09368]) to obtain application solutions with concentrations of 0.005 µg/ml, 0.01 µg/ml, 0.1 µg/ml, 1 µg/ml, 3 µg/ml, 10 µg/ml and 50 µg/ml.

Animals

Female Crl:NMRI mice (*Mus musculus*) were provided by Charles Rivers Laboratories. All animals were housed in isolated ventilated cages under a 12-h light-dark cycle and received food and water *ad libitum*. All animal experimentation was conducted in accordance with German national guidelines and legal regulations.

Application of BI 1291583

*5-day + 2-day treatment holiday regimen*

The animals were treated with BI 1291583 (0.005, 0.05, 0.5 and 5 mg/kg) or vehicle twice daily on Days 1‒5, 8 and 9. BI 1291583 or vehicle was applied orally by gavage (10 ml/kg body weight). On Day 10, animals were treated with BI 1291583 or vehicle followed by an LPS inhalation challenge.

*11-day treatment regimen*

The animals were treated with BI 1291583 (0.00005, 0.0001, 0.001, 0.01, 0.03, 0.1, 0.5, 5 mg/kg) or vehicle once daily for 11 consecutive days. BI 1291583 or vehicle was applied orally by gavage (10 ml/kg body weight). On Day 12, animals were treated with BI 1291583 or vehicle followed by an LPS inhalation challenge.

LPS inhalation

To induce neutrophil influx into the lungs, animals were challenged with an LPS inhalation at Day 12 of this study. LPS from *E.coli* (serotype 055:B5) was used (SigmaAldrich #L2880). A 1 mg/ml LPS solution was prepared in PBS and the animals were challenged with the nebulised solution for 30 min using the MiniHeart Hi-Flo continuous nebuliser (Westmed).

Euthanisation and sample preparation for exposure measurement

Four and a half hours after the LPS challenge, the animals were euthanised by Narcoren application (400–600 mg/kg intraperitoneally) and 5 drops of retrobulbar blood were taken. Plasma was prepared by centrifugation and stored at -20°C for compound exposure measurement. In addition, the femur was prepared and stored at -20°C for exposure measurement in the bone marrow.

Preparation of bronchoalveolar lavage, cell count and differentiation

The trachea was cannulated and a lavage was performed using 2x1 ml lavage buffer (HBSS [HyClone, # Sh30588,01] with 10 U/ml heparin). Cell count in the lavage fluid and cell differentiation were determined using a haemocytometer (Sysmex XT-200i), following the supplier’s instructions.

Preparation of cell lysates

For enzymatic NE measurement, the lavage cells were separated from two 500 µl aliquots of lavage fluid by centrifugation (1500rpm, 4°C, 15min), the supernatants were removed and the pellets were stored at -80°C for further analyses. For enzymatic proteinase 3 (PR3) and Cat G measurement, the remaining lavage samples from all animals of each dose group were pooled and cell count and cell differentiation were determined as described above. Cell pellets were resuspended in lysis buffer (100 mM Tris pH 7.5, 1 M NaCl, 0.2% NP40) and incubated for 10 min on ice. Cell debris was removed by centrifugation (10 min, 1000g, 4°C) and the supernatants were stored at -20°C until further analysis.

Total protein concentration in the lysates was determined using a Pierce™ BCA Protein Assay Kit (ThermoFisherScientific #23225) according to the supplier’s protocol.

Measurement of NE activity

NE activity was measured by the conversion of the fluorescent substrate MeOSuc-AAPV-AMC (Bachem #I-1270) in the cell lysates. The substrate was dissolved in DMSO (50 mM) and a 1 mM stock solution in Tris buffer (100 mM Tris; 1 M NaCl; pH 7.5) was prepared. The cell lysates were diluted to 0.5 mg/ml total protein in buffer (20 mM Tris pH 7.5, 100 mM NaCl, 0.1% HSA).

Cell lysates and blank controls were mixed in a 384-well plate (PerkinElmer #6007270) as follows:

- Blank: 5 µl Tris buffer + 10 µl Tris buffer (+0.1% HSA) + 5 µl NE substrate (1 mM)
- Cell lysate: 5 µl Tris buffer + 10 µl cell lysate + 5 µl NE substrate (1 mM)

Increase in fluorescence was measured over 30 min with a fluorescence reader (spectramaxM5, Molecular devices) with 360 nm excitation wavelength and 460 nm emission wavelength. Fluorescence after 30 min (relative fluorescence units [RFU]) was used to determine NE activity. The mean of the blank values was subtracted from all other values. The blank-corrected RFU value for each sample was then normalised to the number of neutrophils in the sample. A statistical analysis was performed (one-way ANOVA with Dunnett`s multiple comparisons test). Percentage of NE activity in the samples from BI 1291583-treated animals was then calculated relative to the mean of the LPS/vehicle-treated control animals ([NE(compound)/NE(LPSmean)]*100).

ED_50_ was calculated using GraphPad Prism software (version 9.5.0 for Windows, GraphPad Software, www.graphpad.com) with a non-linear regression curve fit. The ED_50_ value was interpolated as the dose of test compound that inhibits 50% of neutrophil elastase activity (relative to the LPS/vehicle-treated control). ED_99_ was calculated from this regression curve using the equation ED_99_ = 99(1/Hill slope)*ED_50_.

Measurement of PR3 activity

PR3 activity was measured by the conversion of the fluorescent substrate Abz-VARCADYQ-EDDnp [1] (custom synthesised by jpt, Innovative Peptide Solutions) in the cell lysates. The substrate was dissolved in DMSO (100 mM) and a 400 µM stock solution in assay buffer (50 mM MES pH 7.5, 700 mM NaCl, 0.06% NP40) was prepared.

Cell lysates and blank controls were mixed in a 384-well plate (PerkinElmer #6007270) as follows:

- Blank: 5 µl assay buffer + 10 µl lysis buffer* + 5 µl PR3 substrate (400 µM)
- Cell lysate: 5 µl assay buffer + 10 µl cell lysate + 5 µl PR3 substrate (400 µM)

Increase in fluorescence was measured over 1 h with a fluorescence reader (spectramaxM5, Molecular devices) with 320 nm excitation wavelength and 420 nm emission wavelength. Fluorescence values after 30 min (RFU) were used to determine PR3 activity. The mean of the blank values was subtracted from all other values. The blank-corrected RFU value for each sample was then normalised to the number of neutrophils in the sample. A statistical analysis was performed (one-way ANOVA with Dunnett`s multiple comparisons test).

Measurement of CatG activity

CatG activity was measured by the conversion of the fluorescent substrate N-Suc-Ala-Ala-Phe-pNA (SigmaAldrich, #S7388) in the cell lysates. The substrate was dissolved in DMSO (20 mM) and an
8 mM stock solution in HEPES buffer (50 mM HEPES pH 7.4, 50 mM NaCl) was prepared. The cell lysates were diluted to 0.9 mg/ml total protein in lysis buffer.

Cell lysates and blank controls were mixed in a 384-well plate (Thermo Scientific NUNC 384-well black, clear bottom; #142761) as follows:

- Blank: 15 µl HEPES buffer + 5 µl CatG substrate (8 mM)
- Cell lysate: 5 µl HEPES buffer + 10 µl cell lysate + 5 µl CatG substrate (8mM)

Increase in fluorescence was measured over 120 min with a fluorescence reader (SpectramaxM5, Molecular devices) with 320 nm excitation wavelength and 405 nm emission wavelength. Enzymatic CatG activity (V_max_) was calculated by the fluorescence reader software (Softmax Pro) and normalised to the number of neutrophils in the sample.

In vivo *inhibition of the production of active NE by* *INS1007*

Methodology was as described for *in vivo* inhibition of the production of NE and PR3 BI 1291583 earlier in this document. However, due to changes to the animal testing licence that occurred during the analysis of INS1007, not all doses were permitted to be tested in one study. Doses were administered in three separate LPS-controlled studies: study 1 received 0.1 mg/kg, 0.3 mg/kg or 0.5 mg/kg; study 2 received 1 mg/kg, 3 mg/kg or 5 mg/kg; study 3 received 5 mg/kg, 30 mg/kg or 50 mg/kg. Head-to-head comparisons of changes in fluorescence for efficacious doses (0.1 mg/kg, 0.5 mg/kg and 5 mg/kg) are not statistically meaningful as the 5 mg/kg dose was administered in separate studies from the study administering 0.1 mg/kg and 0.5 mg/kg doses; therefore, percentage inhibitions compared with LPS control for each study are presented instead.

In vivo *distribution of INS1007*

At approximately 5 hours after final INS1007 administration, animals were euthanised by Narcoren application (400–600 mg/kg intraperitoneally). For assessment of INS1007 exposure in plasma, five drops of retrobulbar blood were taken. For assessment of exposure in the bone marrow, the femur was prepared, epiphysis severed off and bone marrow extracted from the diaphysis and stored at -20°C. Exposure was measured via liquid chromatography-tandem mass spectrometry.

**Results**

*Inhibition of the production of active NE in a neutrophil progenitor cell line by INS1007*

INS1007 inhibited the production of active NE in neutrophil progenitor U937 cells in a concentration-dependent manner, with a mean IC_50_ of 64 nM.

In vivo *inhibition of the production of active NE by INS1007*

After LPS challenge, INS1007 inhibited production of active NE in BALF neutrophils in a dose-dependent manner over the range 0.3 mg/kg–50 mg/kg to a significant (p<0.001) maximum of 76% at 30 mg/kg compared with LPS control (Figure AF3). The ED_50_ value for NE inhibition was calculated at 1.4 mg/kg. At doses matching the efficacious doses of BI 1291583 (0.1 mg/kg, 0.5 mg/kg and 5 mg/kg), INS1007 resulted in no inhibition, a numerical (SEM) 19% (10.4%) and a significant (p<0.001) 75% (4.1%) inhibition of the production of active NE, compared with LPS control. For BI 1291583, these values were significant (p<0.001) 90%, 98% and 99% inhibitions compared with LPS control (Figure AF4).

In vivo *distribution of INS1007*

At approximately 5 hours after the final dose of INS1007 on Day 12, mean (SEM) exposure in bone marrow at doses matching the efficacious doses of BI 1291583 (0.1 mg/kg, 0.5 mg/kg and 5 mg/kg) was 143 (4.1) nM, 321 (15.5) nM and 1693 (123.8) nM, respectively. In plasma, corresponding values were 6 (0.9) nM, 40 (3.1) nM and 710 (71.0) nM. This equates to bone marrow:plasma exposure ratios of 24, 8 and 2, respectively (Figure AF5).

*Kinetic studies of BI 1291583 distribution after a single oral dose in mouse and rat models*

Rapid distribution of BI 1291583 into bone marrow was observed. At 2 hours after a single oral dose of 2 μmol/kg BI 1291583 in a mouse model, mean (SEM) BI 1291583 exposure in bone marrow was 0.12 (0.02) μmol/g. In plasma, the corresponding value was 0.045 (0.016) nM. This equates to a bone marrow to plasma exposure ratio of 2.7. In the rat model, corresponding values were 1.38 (0.50) μmol/g and 0.63 (0.31) nM, yielding an exposure ration of 2.2. BI 1291583 was slowly depleted, with distribution ratios increasing to 148 in mouse at 24 hours and 67 in rat at 48 hours (Figure AF6).

**Table AF1.** BI 1291583 *in vitro* kinetic parameters (surface plasmon resonance), pH 4.5

| **Experiment** | K_on_, M^-1^s^-1^ | K_off_, s^-1^ | K_D_, nM | t_1/2_, minutes* |
| --- | --- | --- | --- | --- |
| 1 | 4.22 x 10^6^ | 1.88 x 10^-3^ | 0.445 | 6.15 |
| 2 | 4.46 x 10^6^ | 2.85 x 10^-3^ | 0.64 | 4.05 |
| 3 | 1.04 x 10^7^ | 2.15 x 10^-3^ | 0.21 | 5.38 |
| Mean | 6.36 x 10^6^ | 2.29 x 10^-3^ | 0.43 | 5.19 |
| SD | 2.86 x 10^6^ | 4.10 x 10^-4^ | 0.18 | 0.87 |

K_D_, equilibrium dissociation constant; K_off_, dissociation rate constant; K_on_, association rate constant; SD, standard deviation; t_1/2_, half-life. * Half-life was calculated by ln_2_/k_off_.

**Table AF2.** Percentage activity of 10 µM BI 1291583 against unrelated proteases

|  | **Inhibition values (%)** | | |
| --- | --- | --- | --- |
| **Protease** | **Assay 1** | **Assay 2** | **Mean** |
| Thrombin | -2.0 | 1.7 | -0.2 |
| Chymase | 2.9 | 13.3 | 8.1 |
| Angiotensin-converting enzyme | -8.9 | -11.6 | -10.2 |
| Angiotensin-converting enzyme-2 | 4.8 | 3.9 | 4.4 |
| Endothelin-converting enzyme | -0.8 | -1.1 | -0.9 |
| Elastase | 0.8 | 2.0 | 1.4 |
| Caspase-1 | 10.7 | 4.7 | 7.7 |
| HIV-1 protease | 7.5 | -1.5 | 3.0 |
| Matrix metalloproteinase-2 | 4.4 | 1.5 | 2.9 |
| Renin | 1.3 | 8.1 | 4.7 |
| Peptidase, kallikrein, plasma | 15.5 | 10.2 | 12.9 |
| Peptidase, matrix metalloproteinase-1 | -0.6 | -1.9 | -1.3 |
| Peptidase, matrix metalloproteinase-3 | 1.7 | -0.7 | 0.5 |
| Peptidase, matrix metalloproteinase- | -0.3 | -0.2 | -0.2 |
| Peptidase, matrix metalloproteinase-8 | 0.1 | 1.4 | 0.7 |
| Peptidase, matrix metalloproteinase-9 | -7.1 | -5.8 | -6.4 |
| Peptidase, matrix metalloproteinase-12 | -6.2 | -6.6 | -6.4 |
| Peptidase, matrix metalloproteinase-13 | -1.8 | -1.3 | -1.6 |
| Peptidase, matrix metalloproteinase-1 | 1.7 | -2.5 | -0.4 |
| Peptidase, PLAU (urokinase) | 1.2 | 0.8 | 0.9 |
| Peptidase, caspase 2 | -1.4 | 0.7 | -0.4 |
| Peptidase, caspase 3 | 3.8 | 6.9 | 5.3 |
| Peptidase, CASP6 caspase 6 | 0.2 | 4.2 | 2.2 |
| Peptidase, caspase 7 | -1.3 | -1.2 | -1.3 |
| Peptidase, caspase 8 | 2.2 | 2.5 | 2.4 |
| Peptidase, caspase 9 | 0.7 | -0.6 | 0.1 |
| Peptidase, caspase 10 | 7.9 | 9.1 | 8.5 |
| Peptidase, metalloproteinase, neutral endopeptidase | 2.0 | -0.4 | 0.8 |
| Peptidase, beta-secretase | 13.4 | 10.4 | 11.9 |
| Peptidase, trypsin | -5.8 | -2.7 | -4.2 |
| Peptidase, tryptase | -6.3 | -5.7 | -6.0 |
| Peptidase, tumour necrosis factor-α-converting enzyme | 2.1 | 2.4 | 2.3 |
| Peptidase, neutrophil elastase 2 | -0.6 | -5.6 | -3.1 |

**Figure AF1.** A. NE and B. CatG activity in mouse BALF neutrophil lysate after treatment with BI 1291583 and subsequent LPS challenge, 5+2-day dosing


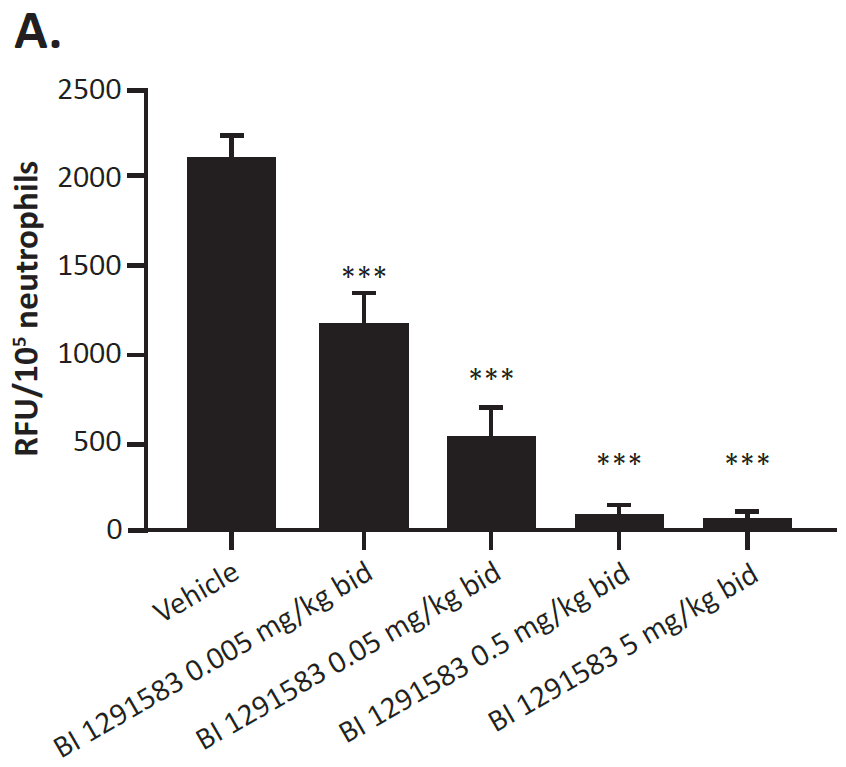


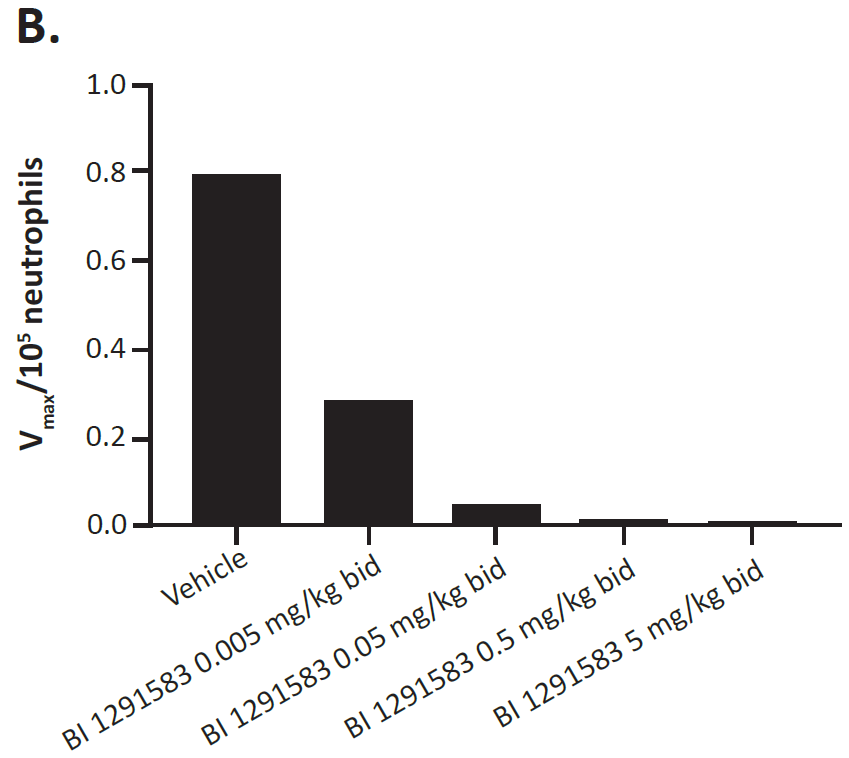


BALF, bronchoalveolar lavage fluid; bid, twice daily; CatG, cathepsin G; LPS, lipopolysaccharide; NE, neutrophil elastase; RFU, relative fluorescence units; V_max_, reaction rate when CatC is saturated with CatG. *** p<0.001 compared with vehicle. Samples were pooled for analysis of CatG activity. In A, data are mean and error bars indicate standard error of the mean.


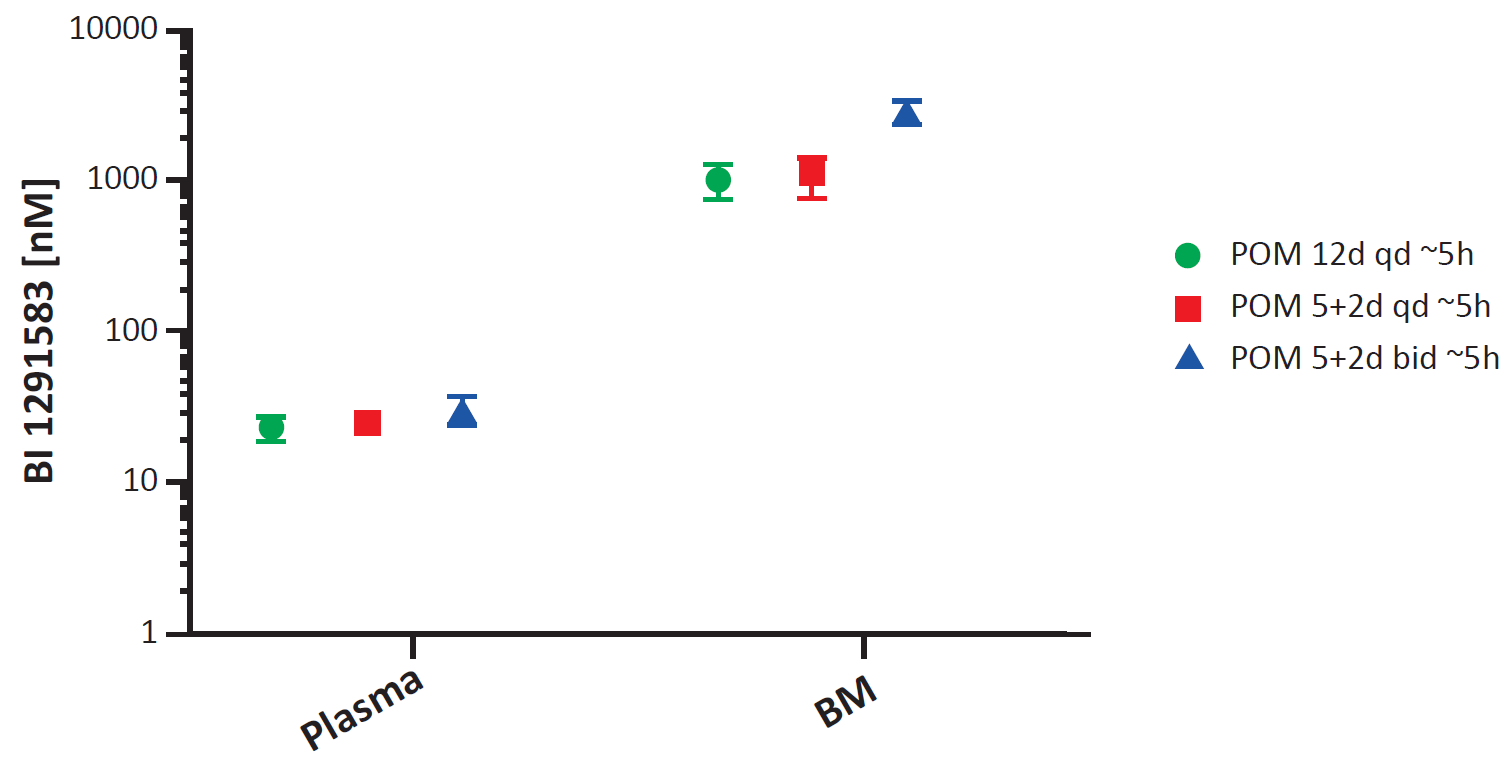
**Figure AF2.** Distribution of BI 1291583 at approximately 5 hours after 5+2-day and 12-day 0.5 mg/kg bid dosing *in vivo*

bid, twice daily; BM, bone marrow; POM, proof-of-mechanism model; qd, once daily. Data are mean. Error bars indicate standard error of the mean.

**Figure AF3.**  NE activity in mouse BAL neutrophil lysate after treatment with INS1007 and subsequent LPS challenge, 11-day dosing


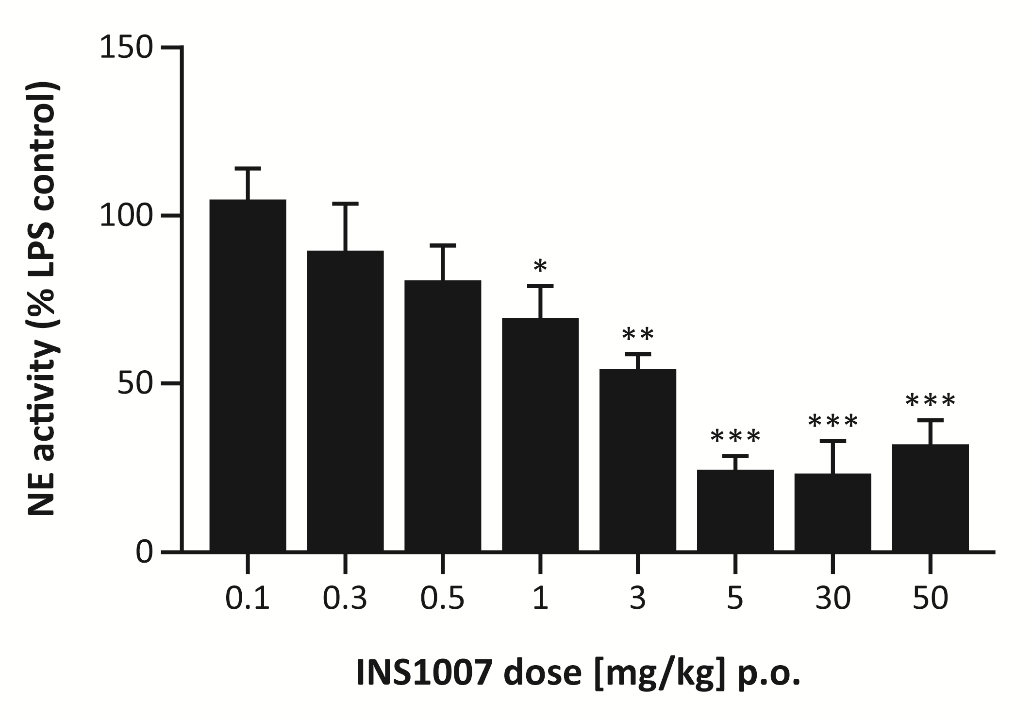


BAL, bronchoalveolar lavage; LPS, lipopolysaccharide; NE, neutrophil elastase; p.o., orally.
* p<0.05; ** p<0.005; *** p<0.001 compared with vehicle. Data are mean. Error bars indicate standard error of the mean.

**
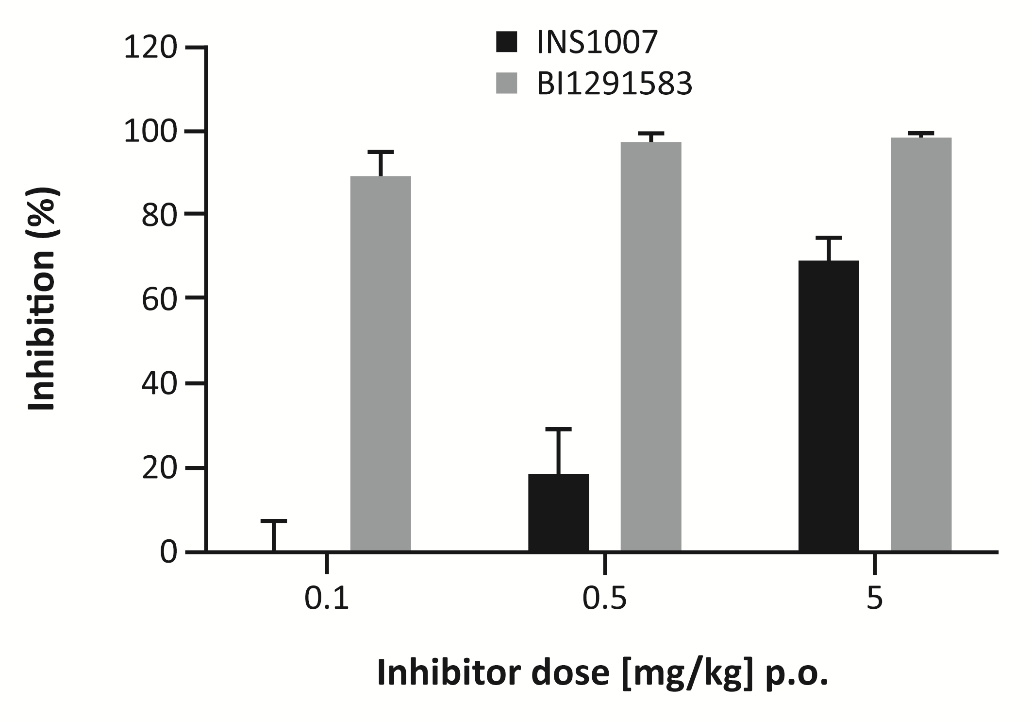
Figure AF4.** Comparison of NE inhibition in mouse BAL neutrophil lysate after treatment with

BI 1291583 or INS1007 and subsequent LPS challenge, 11-day dosing

BAL, bronchoalveolar lavage; LPS, lipopolysaccharide; NE, neutrophil elastase; p.o., orally. Data are mean. Error bars indicate standard error of the mean.

**
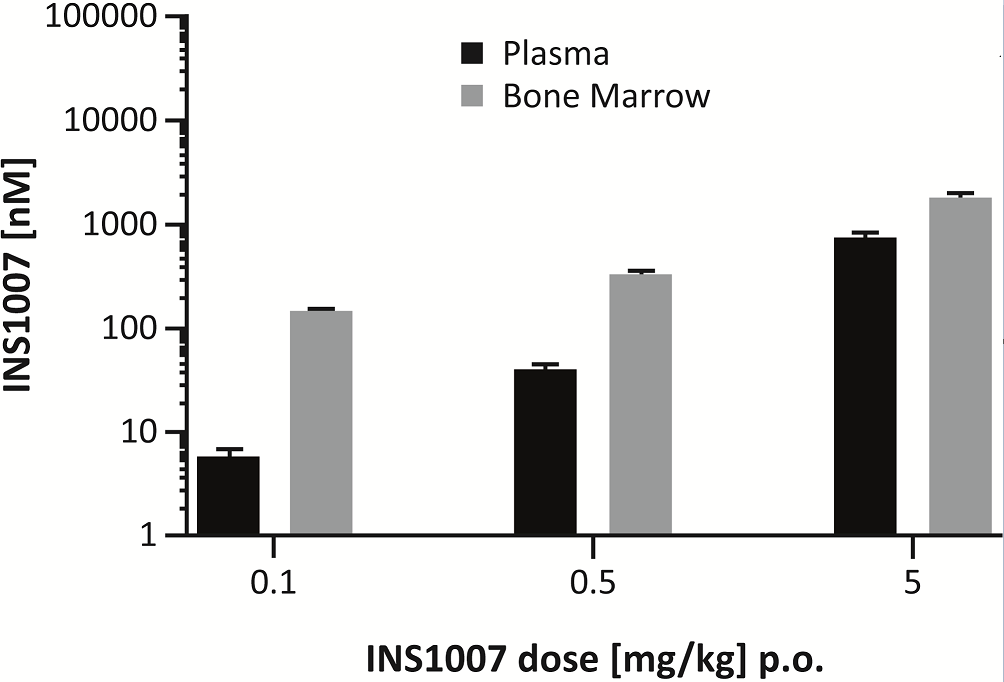
Figure AF5.** Bone marrow and plasma distribution of INS1007 at approximately 5 hours post-administration, 11-day dosing

p.o., orally. Data are mean. Error bars indicate standard error of the mean.

**
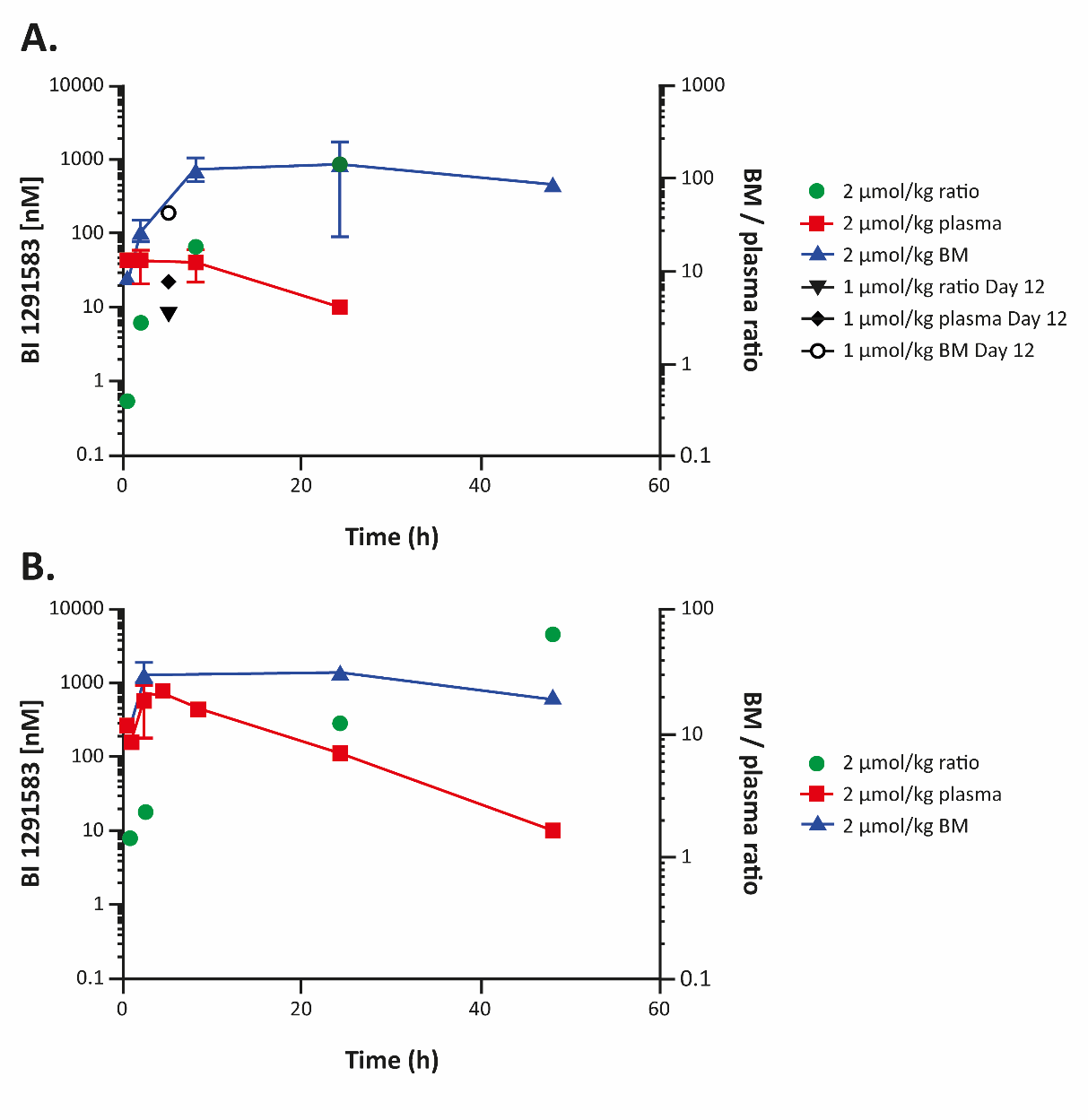
Figure AF6.** Time-dependent bone marrow and plasma BI 1291583 exposure in A) mouse and B) rat models

BM, bone marrow. Data are mean. Error bars indicate standard error of the mean over duplicate experiments.

**References**

1. Kalupov T, Brillard-Bourdet M, Dadé S, Serrano H, Wartelle J, Guyot N, et al. Structural characterization of mouse neutrophil serine proteases and identification of their substrate specificities: relevance to mouse models of human inflammatory diseases. J Biol Chem. 2009;284:34084-91.
